# *Pantoea agglomerans* in Immunodeficient Patients with Different Respiratory Symptoms

**DOI:** 10.1100/2012/156827

**Published:** 2012-04-24

**Authors:** Erika Odilia Flores Popoca, Maximino Miranda García, Socorro Romero Figueroa, Aurelio Mendoza Medellín, Horacio Sandoval Trujillo, Hilda Victoria Silva Rojas, Ninfa Ramírez Durán

**Affiliations:** ^1^Programa de Doctorado en Ciencias de la Salud, Facultad de Medicina, Universidad Autónoma del Estado de México, 50180 Toluca, MEX, Mexico; ^2^Clínica de Epidemiologia, Hospital General de Zona 194, IMSS, 06720 México, DF, Mexico; ^3^Coordinación de Investigación, Delegacion Estado de México, IMSS, 5000 Toluca, MEX, Mexico; ^4^Departamento de Sistemas Biológicos, Universidad Autónoma Metropolitana Xochimilco, 04960 México, DF, Mexico; ^5^Colegio de Postgraduados, Campus Montecillo, 56230 Texcoco, MEX, Mexico

## Abstract

The aim of this paper was to determine in 32 patients from 4 different Mexican hospitals the frequency of opportunistic bacteria in the 2010 to 2011 time period. The patients were divided in 4 groups. Group 1 included 21 HIV positive patients with acute respiratory syndrome. Four HIV positive patients with tuberculosis symptoms were included in Group 2; two patients with tuberculosis symptoms and one asymptomatic person formed Group 3. Reference Group 4 included 4 patients from whom 4 strains of *Mycobacterium* spp. had been reported. The strains were isolated and identified by 16S rRNA gene amplification, API 20E and 50CH, biochemical test, and antibiotic sensitivity. The strains found were 10 *Pantoea agglomerans*, 6 *Mycobacterium* spp., 6 *Pseudomonas* spp. and 10 strains of normal floral species: *Thermoactinomycetes bacterium* (1), *Enterococcus faecium* (2), *Bacillus licheniformis* (1), *Lactobacillus rhamnosus* (2), *Streptococcus oralis* (2), *Streptococcus anginosus* (1), and *Enterobacter hormaechei* (1).

## 1. Introduction

 Immunodeficiency is the main conditioning factor for bacterial infection susceptibility; therefore, immunodeficient patients frequently present several aggregated infections [1]. Since the eighties there has been an increase in opportunistic microorganisms in addition to the human immunodeficiency virus (HIV positive) [2]. Some diseases such as tuberculosis (TB) are related to HIV positive infection but the symptoms and signs are nonspecific and it is difficult to distinguish TB from other opportunistic infections. The patient may have acute symptoms for a few hours or days or fever and nonspecific systemic symptoms for several days or weeks similar to bacterial infection (or weight loss) [3, 4].

It has been established that worldwide 60 million people have active TB and 2-3 million die yearly due to *Mycobacterium tuberculosis. *The complex diagnosis of respiratory symptoms leads to believe that other opportunistic bacteria such as *P. agglomerans* are involved in immunodeficient patients [3]. *P. agglomerans *is a Gram negative bacillus of the *Enterobacteriaceae* family. It is ubiquitous and found in humans, animals, and plants [5, 6], soil, and water, and it is an important microorganism from the medical point of view [7]. It is often opportunistic and requires an immunodeficient host for its growth [8].

This bacterium causes infection and is frequently associated to other conventional pathogens [9]. In the diagnosis of infectious diseases and identification of the causal agent, the molecular biology methods are useful. The comparative sequencing of the 16 s rRNA gene confirms the presence of opportunistic microorganisms in immnodeficient patients [10].

The objective of this research was to identify the predominant bacterial species in HIV positive and other immunodeficient patients with respiratory symptoms and with or without a TB diagnosis.

## 2. Materials and Methods

### 2.1. Patients

The study included 32 patients from four hospitals in the State of Mexico, during the years 2010-2011, and were divided into four groups.

**Group 1: HIV Positive Patients with Acute Respiratory Syndrome**

Twenty-one HIV positive patients with acute respiratory syndrome, 16 males and 5 females, aged 31 to 54 years, came from the “Acquired Inmunedeficiency Syndrome (AIDS)” clinic of a main hospital. They presented 41 to 958 CD4 values and <40–110 viral load. They also presented respiratory symptoms, coughing, expectoration, and a 3–20 kg weight loss suggestive of TB. No laboratory tests to confirm the diagnoses were carried out until this study began but the patients received antiretroviral treatment.

**Group 2: HIV Positive Patients with TB Syndrome**

Four HIV positive patients with TB syndrome, 3 males and one female 31–37 years old, with 288–576 CD4 values and no detectable viral load were admitted to a specialized AIDS and sexual transmitted disease hospital, with a diagnoses suggestive of TB (cough, expectoration, fever, and a 5–13 kg weight loss). The diagnosis was confirmed by bacilloscopy and the patients received a doTBal shortened strictly supervised tuberculosis treatment (TAES) rifampicin, isoniazid, 3 pyrazinamide, and ethambutol chloride, for six months.

**Group 3: Patients with a TB Syndrome**

Two patients with a TB syndrome, a 32-years-old male and a 39-year-old female, from an AIDS and sexually transmitted diseases clinic, presented cough, expectoration, fever, and 5 and 10 kg weight loss, respectively, and were diagnosed with TB after positive bacilloscopy. They received a doTBal shortened strictly supervised treatment (TAES) (rifampicin, isoniazid, pyrazinamide, and ethambutol chloride for six months. A sputum sample was obtained from a 30-year-old male companion without symptoms or diagnosis but with laboratory diagnostic tests. He was considered as an asymptomatic high-risk patient.

**Group 4: Reference Group**

Four patients were located from a retrospective study (2007–2010) of 186 clinical files of a specialized hospital to detect the number of patients with TB and the most common* Mycobacterium *strain in the State of Mexico. This hospital provided four bacterial strains, already isolated, from the four patients and identified as *Mycobacterium* spp. by means of the Polymerase Chain Reaction (PCR) technique. The difference between Group 3 and Group 4 is that strains of Group 3 were isolated by us and strains of Group 4 were provided already isolated by the hospital's laboratory staff and patients were not available.

### 2.2. Specimen Procurement and Processing

In accordance with the guideline for tuberculosis bacteriological diagnoses (MDBT) [11, 12], the 28 patients from Groups 1–3 were interviewed while fasting and asked for a sputum sample for 3 consecutive days. The specimens were collected in sterile flasks and processed at the Medical and Environmental Microbiology Laboratory of the Faculty of Medicine at the Autonomous University of the State of Mexico, Mexico, and were analyzed in accordance with the guideline for TB bacteriological diagnoses. The specimens were submitted to a digestive liquefaction and decontamination process with alkaline N-acethyl-L-cysteine in order to eliminate bacteria that interfere with *Mycobacterium* and to liquefy tissue, mucus, and other organic materials [11, 12].

### 2.3. Culturing and Isolation

Decontaminated samples were put into in commercial Lowenstein-Jensen (LJ) (BD BBL 0196675) medium, incubated at 37°C for 3-4 weeks, and revised every 7 days. The cultured bacteria were transferred to Petri plates containing Middlebrook 7H10 Agar (Difco, USA) glycerol and OADC enrichment culture medium and incubated at 37°C [11, 12]. The 32 strains were cultured in the Middlebrook liquid medium to obtain biomass for DNA extraction.

Twenty-eight strains from the patients of Groups 1, 2, and 3 were obtained considering the colony morphology: size, color, surface, and consistency, and 4 strains from the patients in the reference group were acquired from the IMSS Medical Center “Siglo XXl” Tuberculosis Service. The chosen bacteria were smeared, the slides were stained with Ziehl-Neelsen for acid-alcohol resistant bacilli (BAAR) [5, 13], and a CME Microscope 1349521X (Leica, USA) was used.

### 2.4. Genetic Characterization

For total DNA extraction the Wizard Genomic Purification DNA Kit (Promega A1120) (Isolation of genomic DNA from Gram positive and Gram negative bacteria) was used. For PCR amplifications and sequencing, A 1300–1500 bp fragment of the 16S rRNA gene was amplified with primers 8F (5′-AGA GTT TGA TCA TGG CTC AG-3′) and 1492R (5′-TAC GGT TAC CTT GTT ACG ACT T-3′). After 30 cycles of denaturation at 94°C for 60 s, primer annealing at 55°C for 20 s, and primer extension at 72°C for 60 s, followed by postamplification extension at 72°C for 10 min, amplification was seen in (1%) agarose gel electrophoresis at 90 volts for 30 min. The amplified products were sequenced for Bagdye terminator to Macrogen USA. Sequences corresponding to both regions were assembled and edited using BioEdit software version 7.0.5 [14] and a consensus sequence of each isolate was created. Since the Basic Local Alignment Search Tool (BLASTN) from the NCBI finds regions of local similarity between sequences with significant alignments, consensus sequences of each isolate obtained for 16S rDNA partial sequence were submitted to BLASTN 2.2.25 [15].

### 2.5. Predominant Strains

The identified predominant group of strains were subjected to phylogenetic and biochemical characterization and to antibiotic sensibility studies.

### 2.6. Phylogeny

All consensus sequences were compiled into a single file (Fasta format) for evolutionary analyses and then aligned with the profile mode of Clustal W 1.8.1 [16] included in Mega 4.0.2 software [17]. Phylogeny reconstruction analysis was performed with a Maximum Parsimony method on the *P. agglomerans* dataset. This analysis was performed using search options Close Neighbor Interchange (CNI) search (level = 1), with an initial tree by random addition (10 reps); gap/missing data were considered as a complete deletion.

### 2.7. Biochemical Characterization

For the biochemical characterization each colony was identified using the API 20E (Biomérieux B-20100) and API 50CH profile index (Biomérieux B-50300). The systems were incubated at 35°C and all reactions were read after 20 to 24 h. Results of affirmative or negative tests were recorded and interpreted according to the manufacturer's directions [18].

### 2.8. Antibiotic Sensitivity

For antibiotic sensitivity the strains were cultured in Mueller-Hinton Agar (MH) (Bioxon, U.S.A); antimicrobial susceptibility was determined with the disk diffusion method accordingly to the National Committee for Clinical Laboratory Standards (NCCLS) [19]. The bacteria were classified as resistant (R), intermediate (I), and susceptible (S) to a particular antimicrobial agent. The antibiotics used were amikacin, ampicillin, carbenicillin, cephalothin, cefotaxime, ceftriaxone, chloramphenicol, gentamicin, netilmicin, nitrofurantoin, pefloxacin, and trimetoprim-sulfametoxazol. Petri dishes containing the antibiotic disks were inoculated and incubated at 35°C for 24 h. Resistance or susceptibility to any of the antibiotics was determined from the halo diameter and data found in tables [19].

## 3. Results

The demographic characteristics of the patients, the CD4 values, and viral load are shown in Table 1.

### 3.1. Bacilloscopy

Of the 28 sputum specimens, 12 were BAAR positive: five strains from Group 1 patients, four strains from Group 2, and three strains from Group 3 patients. All the isolates showed good growth in the two culture media (LJ and Middlebrook 7H10). The acid-fast smear microscopy results (>90%) were given to the health care provider within 24 hours of receipt of all specimens.

### 3.2. Macroscopic and Microscopic Morphology of the Bacteria

The macroscopic and microscopic morphology of the bacteria allowed grouping in four categories. Category A included small size, yellow, round, and creamy like colonies. Ten strains were short bacilli, with round ends, Gram negative, and no BAAR. Category B was small size, dark yellow, round, and wrinkled colonies. In 6 strains the bacilli were elongated, Gram positive, and acid-alcohol resistant. Category C included medium size, cream and yellow color, round, and creamy colonies. Six strains showed straight or slightly curved bacilli, Gram negative, and no BAAR. Category D included a variety of small to medium size, yellow and cream color, smooth, hard, and creamy colonies. Ten strains were of Gram positive diplococci and Gram positive bacilli, no BAAR. The acid-fast smear microscopy results (>90%) were given to the health care provider within 24 hours of receipt of all specimens.

### 3.3. Identification

With the comparative analysis of the obtained sequences deposited in Gen Bank, the principal species were identified: ten strains were identified as *P. agglomerans* corresponding to Category A; six strains corresponding to Category B were identified as* Mycobacterium *spp. (2 *M. tuberculosis *y 4 *M. parascrofulaceum*); six strains corresponding to Category C were* Pseudomonas *spp. (1 strain was *P. koreensis*, 4 strains were *P. azelaica,* and one was *P. aerogenes*); in the D category 10 strains of the normal flora were identified as species *Thermoactinomycetes bacterium *(1),* Enterococcus faecium* (2), *Bacillus licheniformis *(1)*, Lactobacillus rhamnosus *(2)*, Streptococcus oralis *(2)*, Streptococcus anginosus *(1), and *Enterobacter hormaechei* (1). The distribution and percentage of the strains in their Categories are shown in Table 2.

### 3.4. Phylogeny

**Figure 1:**
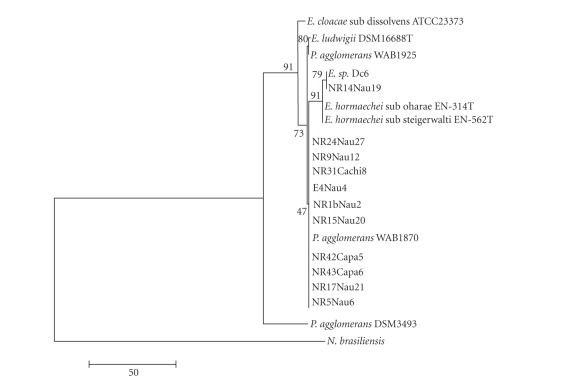
Phylogenetic tree based on 1367 bp without gaps from 16S rRNA gene. *Nocardia brasiliensis* was used as the out-group of phylogenetic tree.

The analysis of the sequencing of fragment 1367 bp without gaps from 16S rRNA gene was carried out by comparison with five species of the genus *Enterobacter* and three species of the genus *Pantoea,* and the sequence of *Nocardia brasiliensis* was used to form the roots of the tree. The ten analysed strains were grouped with the *P. agglomerans* sequence with 91% bootstrap value (Figure 1).

### 3.5. Biochemical Characterization by API 20E and API 50CH Test Strips

Since *P. agglomerans* predominated (31.25%), it underwent phylogeny studies and antibiotic sensibility assays. The ten *P. agglomerans* strains were biochemical characterized by API 20E and API 50CH test strips. All had the same profile with both systems indicating that are aerobic bacteria and ferment: B-galactose, lactose, saccharose, ornithine, glucose, arabinose, arginine, mannitol, inositol, sorbitol, rhamnose, melibiose, and amygdaline. Voges Proskauer was positive and, therefore, produced buthyenlglicolic fermentation. They are citrate positive and indol negative, do not produce sulphydric acid or urease, do not produce desamination of tryptophan substrate or urease, and do not catabolize lysine and the gelatinase test was negative.

### 3.6. Antimicrobial Susceptibility

Most of the *P. agglomerans *were resistant to carbeniillin, ceftriaxone, chloramphenicol, gentamycin, netilmicin, nitrofurantoin, and pefloxacin. Some strains were resistant to amikacin, ampicillin, and ctoyoxin (Table 3).

## 4. Discussion

The symptoms of the included patients are similar to those produced by *Mycobacterium *spp. Nevertheless, the presence of two or more causes for pulmonary disease is also characteristic of HIV positive patients who frequently present with bacterial infections, an important cause for high morbility and mortality. Some researchers have indicated that this situation occurs in 39% of the patients [20] and others have established that nosocomial infections increase in 5% a fatal risk [21].

García et al. (2003) reported that 100% of the patients admitted in a hospital present with pulmonary diseases mostly due to opportunistic infections (98.7%), and in HIV positive patients, the 3 principal causes were, TB, bacterial pneumonia and other no specified infectious neumonia [22].

The results of this investigation indicate that not all respiratory syndrome in immunodeficient patients is due to TB. The patients studied presented with productive cough but the identified organisms were *P. agglomerans, Mycobacterium, *and* Pseudomonas. *These microorganisms are considered opportunists and the differential diagnosis is necessary for the proper treatment before other organs are affected.

The mostly isolated bacterium was *P. agglomerans, *a Gram negative bacillus with predilection for the lungs. According to Rodríguez and Martínez (2002), nosocomial infections in adult patients are caused by Gram negative bacilli, mainly enterobacteria [23], a fact that agrees with Kaye (2001) who isolated mostly *P. agglomerans, *in respiratory secretions [24].

On the other hand, Domínguez et al. in 2008 reported that the most frequently isolated Gram negative bacillus is *Pseudomonas *spp. followed by *Enterobacter*. The low number of lymphocytes is a factor that increases the risk of these infections [25]. In a 2005 study by Chernilo et al., there were 14 nosocomial pneumonia and in the sputum were isolated several infectious agents:* P. aeruginosa* (two specimens) and *Enterobacter* in one sample [26].

In general, *Mycobacterium *spp. presence was discarded in Group 1 of patients but other opportunistic bacteria were found which in HIV positive patients can be a health risk.

In Group 2 we expected to isolate *Mycobacterium* spp. since these patients had the clinical symptoms and the bacterium was confirmed, but instead, *P. agglomerans *and *Pseudomonas* spp. were found. This might have been because the anti-TB treatment eliminated *Mycobacterium *and other types of bacteria flourished in these HIV positive patients.

As to the patients in Group 3, it is interesting that their sputum was negative for Ziehl-Neelsen but culture positive. This result implies an epidemiologic factor. The related persons in contact with the TB patient are acid-fast bacteria negative and are sent home without diagnosis or treatment but they are a focus of infection for other persons.

In the present study, we confirmed bacterial identification by means of the amplified sequence of 16S rRNA gene. Molecular biology techniques are a useful tool for the confirmation of the isolated organism, as is the case of *P. agglomerans, *a seldom mortal microorganism frequently considered as opportunistic, since the symptoms can be confused with those caused by* Mycobacterium* spp. if not correctly identified.

By means of the API 20E and 50CH biochemical assays practiced on *P. agglomerans, *its biochemical and metabolic characteristics were determined. This result might be very useful to complement the identification with a phenotypic study. The resistance and sensibility of *P. agglomerans *to the most frequent antimicrobial agents for Gram negative bacteria were determined.

## 5. Conclusions

From the 32 included patients, the following strains were identified: ten strains of *P. agglomerans* were in Category A; 6 strains of *Mycobacterium* spp. (two *M. tuberculosis* and 4 *M. parascrofulaceum*) were in Category B; six strains of *Pseudomonas *spp. (one strain of *P. koreensis*, one *P. aerogenes*, and 4 strains of *P. azelaica*) were found in patients in the Category C. In the Category D ten strains of normal flora were isolated.

Even though, initially the aim our study was the search and identification of *Mycobacterium* spp. The most frequently identified species found was *P. agglomerans *(31.25%) followed by *Mycobacterium* spp. (18.75%) and *Pseudomonas* spp. (18.75%) and several normal flora bacteria represent 31.25%.

Our research confirms the presence of opportunistic bacteria such as *P. agglomerans* in HIV positive and other immunodeficient patients with clinical respiratory symptoms related to TB (*Mycobacterium* spp.), a situation that has to be accounted for in the correct diagnosis and patient follow-up.

## Figures and Tables

**Table 1:** Demographic characteristics of the patients, CD4 values and viral load.

Patients	Code	Male/female	Age	Weight loss (kg)	Values of CD4	Viral load
Group 1. HIV positive patients with acute respiratory syndrome						

(1)	E1	M	51	3	47	77
(2)	NR1b	M	39	20	850	<40
(3)	E2	F	39	6	395	60
(4)	E4	M	45	6	958	<40
(5)	NR5	F	44	6	837	<40
(6)	NR6	M	39	5	377	<40
(7)	NR7	M	43	6	ND	ND
(8)	NR8	M	36	6	329	<40
(9)	NR9	M	35	10	267	<40
(10)	NR10	M	40	7	41	<40
(11)	E6	M	31	10	ND	ND
(12)	NR14	M	45	8	316	<40
(13)	NR15	M	31	4	579	<40
(14)	NR17	M	37	7	814	82
(15)	NR18	F	54	Without loss	ND	ND
(16)	NR19	F	41	15	809	<40
(17)	NR21	M	30	10	492	111
(18)	NR22	M	27	3	474	66
(19)	NR23	F	35	16	662	<40
(20)	NR24	M	32	18	ND	ND
(21)	NR25	M	31	3	623	<40

Group 2. HIV positive patients with TB syndrome						

(1)	E9	M	35	10	288	Indetectable
(2)	NR41	M	31	5	345	Indetectable
(3)	NR42	F	26	Without loss	576	Indetectable
(4)	NR43	M	37	13	438	Indetectable

Group 3. Patients with TB syndrome						

(1)	NR36	M	32	5	No	No
(2)	NR31	F	39	10	No	No
(3)	NR37	M	30	Without loss	No	No

Group 4. Reference group						

(1)	NR51	M	51	Without loss	No	No
(2)	NR52	F	51	Without loss	No	No
(3)	NR53	M	81	2	No	No
(4)	NR54	F	85	5	No	No

ND: No determined; NO: no CD4 values/no viral load determined.

**Table 2:** Sequenced identified bacteria and the similarity percentage calculated with the BLASTN program.

Patients	Code	BLASTN program	Morphology group	Similarity percentage
Group 1. HIV positive patients with acute respiratory syndrome				

(1)	E1	*Thermoactinomycetes bacterium*	Category D	99
(2)	NR1b	*Pantoea agglomerans*	Category A	100
(3)	E2	*Enterococcus faecium*	Category D	100
(4)	E4	*Pantoea agglomerans*	Category A	100
(5)	NR5	*Pantoea agglomerans*	Category A	99
(6)	NR6	*Bacillus licheniformis*	Category B	99
(7)	NR7	*Lactobacillus rhamnosus*	Category D	99
(8)	NR8	*Streptococcus oralis*	Category D	99
(9)	NR9	*Pantoea agglomerans*	Category A	99
(10)	NR10	*Streptococcus anginosus*	Category D	99
(11)	E6	*Lactobacillus rhamnosus*	Category D	97
(12)	NR14	*Enterobacter hormaechei*	Category D	99
(13)	NR15	*Pantoea agglomerans*	Category A	99
(14)	NR17	*Pantoea agglomerans*	Category A	100
(15)	NR18	*Enterococcus faecium*	Category D	97
(16)	NR19	*Pseudomonas koreensis*	Category C	99
(17)	NR21	*Pseudomonas azelaica*	Category C	99
(18)	NR22	*Pseudomonas azelaica*	Category C	99
(19)	NR23	*Pseudomonas azelaica*	Category C	99
(20)	NR24	*Pantoea agglomerans*	Category A	99
(21)	NR25	*Streptococcus oralis*	Category E	99

Group 2. HIV positive patients with TB syndrome				

(1)	E9	*Pseudomonas aeruginosa*	Category C	99
(2)	NR41	*Pseudomonas azelaica*	Category C	99
(3)	NR42	*Pantoea agglomerans*	Category A	99
(4)	NR43	*Pantoea agglomerans*	Category A	100

Group 3. Patients with a TB syndrome				

(1)	NR36	*Mycobacterium tuberculosis*	Category B	100
(2)	NR31	*Pantoea agglomerans*	Category A	99
(3)	NR37	*Mycobacterium tuberculosis*	Category B	100

Group 4. Reference group				

(1)	NR51	*Mycobacterium parascrofulaceum*	Category B	97
(2)	NR52	*Mycobacterium parascrofulaceum*	Category B	99
(3)	NR53	*Mycobacterium parascrofulaceum*	Category B	99
(4)	NR54	*Mycobacterium parascrofulaceum*	Category B	99

**Table 3:** Antibiogram results for microbial.

Antibiotic	NR5	NR9	N17	NR24	NR43	NR31
Amikacin (AK)	R	I	R	S	S	R
Ampicillin (AM)	S	R	R	R	R	R
Carbenicillin (CB)	R	R	R	R	R	R
Cefotaxima (CTX)	S	R	R	I	R	R
Ceftriaxone (CRO)	R	R	R	I	R	R
Choramphenicol (CL)	R	I	I	R	R	I
Gentamycin (GE)	R	I	I	R	S	R
Netilmicin (NET)	R	R	R	R	R	R
Nitrofurantoin (NF)	I	R	R	R	R	R
Pefloxacin (PEF)	R	R	R	R	R	R

R: Resistant; I: Intermediate; S: Susceptible.
